# Association of Ulcerative Colitis with *FOXP3* Gene Polymorphisms and Its Colonic Expression in Chinese Patients

**DOI:** 10.1155/2019/4052168

**Published:** 2019-02-24

**Authors:** Sheng-Long Xia, Shi-Jie Ying, Qian-Ru Lin, Xiao-Qi Wang, Wei-Jun Hong, Zi-Jian Lin, Jia-Kai Luo, Yi Jiang

**Affiliations:** Department of Gastroenterology, The Second Affiliated Hospital of Wenzhou Medical University, 109 Xueyuan West Road, Wenzhou, 325000 Zhejiang Province, China

## Abstract

Abnormalities of forkhead box P3 (FOXP3) are implicated in various autoimmune diseases. This study is aimed at investigating the association of ulcerative colitis (UC) with *FOXP3* polymorphisms and its colonic expression in Chinese patients. Polymorphisms of rs3761548, rs2232365, rs2294021, and rs3761547 were examined in 472 UC patients and 525 healthy controls using the SNaPshot method. The colonic expression of *FOXP3* mRNA and protein was assayed in inflammatory mucosa of 34 UC patients and normal mucosa of 36 patients with benign sigmoid polyps (normal controls) using real-time quantitative polymerase chain reaction and immunohistochemical analysis. All data were handled separately for females and males. As a result, the carrier frequencies with at least one variant allele of rs3761548, rs2232365, and rs229402 increased in female and male UC patients compared with healthy controls. Significant differences in these carrier frequencies were also observed between patients with mild and moderate UC and patients with severe UC. The expression of *FOXP3* was higher in UC patients (both males and females), especially those with severe UC, than in normal controls. The expression of *FOXP3* was downregulated in UC patients having at least one variant allele compared with UC patients having no variant allele of rs3761548, rs2232365, and rs2294021. Male gender (*β* = −0.341), rs2294021 variation (*β* = −0.503), and severe UC (*β* = 0.361) were independently related to the mRNA expression of *FOXP3* in UC patients. Together, our findings indicated that *FOXP3* (rs3761548, rs2232365, and rs2294021) variations increased the risk of UC and were associated with the lower colonic expression of *FOXP3* in UC patients.

## 1. Introduction

Regulatory T cells (Tregs), especially those that coexpress CD4 and CD25, are crucial in the suppression of immune response and orchestration of immune tolerance. Several lines of evidence suggest that Tregs may modulate potentially self-reactive T cells through the secretion of suppressive cytokines [e.g., interleukin- (IL-) 10 and IL-35], expression of inhibitory receptors, and direct cell-contact-dependent mechanisms [1, 2]. The numerical or functional abnormality of Tregs is further confirmed to cause a breach in self-tolerance and hence is closely involved in a range of immune-related diseases, including systemic lupus erythematosus (SLE), rheumatoid arthritis (RA), multiple sclerosis (MS), and inflammatory bowel disease (IBD). IBD mainly comprises two phenotypes: ulcerative colitis (UC) and Crohn's disease (CD) [3, 4]. Maul et al. revealed that the number of Tregs was higher in the inflamed mucosa of patients with IBD than in healthy individuals, but significantly lower compared with other inflammatory conditions, such as diverticulitis [5]. Notably, antitumor necrosis factor treatment, a well-described and effective approach to treating IBD, has been demonstrated to facilitate the increase in the number of Tregs in the circulation and colonic mucosa of patients with IBD [6, 7], highlighting that dysregulation of Tregs may have an impact on the progression of IBD.

As a member of the forkhead–winged helix family of transcription regulators, forkhead box P3 (FOXP3) is identified to be a lineage-specifying factor responsible for the development, lineage commitment, and regulatory functions of Tregs. The current study confirmed that FOXP3 might regulate the expression of critical immunosuppressive molecules via binding to their relative target genes, such as cytotoxic T-lymphocyte antigen 4 (CTLA4) [8]. In addition, FOXP3 can also promote the conversion of naïve T cells to Treg-like cells with suppression activity and affect the differentiation and function of Tregs [8–10]. Hori et al. previously noted that the ectopic expression of *FOXP3* in CD4^+^ CD25^–^ T cells was able to endow a Treg phenotype to prevent IBD [11]. Conversely, the downregulation or deletion of the expression of *FOXP3* was shown to result in an impaired Treg function [12]. Numerous studies to date have implicated the aberrant expression of FOXP3 in multiple immune-related diseases, such as SLE, RA, and MS [10].

The human *FOXP3* gene, which contains 12 exons and 11 introns, is mapped on the small arm of the X chromosome (p11.23–13.3) [13]. The existing literature shows that some polymorphisms in the promoter region of *FOXP*3 can potentially modify gene expression by changing the sequence of transcription factor-binding sites and/or by modulating the kinetics of the transcription process [9]. For example, rs3761548 was reported to have functional consequences and influence the inhibitory function of Tregs, ultimately increasing vulnerability to Graves' disease (GD) in a Chinese population [14]. This study focused on four single-nucleotide polymorphisms (SNPs): rs3761548 (-3279, C/A), rs2232365 (-924, A/G), rs2294021 (C/T), and rs3761547 (-3499, C/A), which are positioned at or near the promoter region of *FOXP*3. Importantly, previous studies have demonstrated that the four SNPs are the most common polymorphic loci of *FOXP*3 in an Asian population [15–19]. Besides, the expression of *FOXP3* mRNA and protein in colonic tissues was examined to address the association of UC with *FOXP3* polymorphisms and its colonic expression in this cohort of Chinese patients.

## 2. Materials and Methods

### 2.1. Study Subjects

A total of 472 patients with UC and 525 healthy individuals were recruited from January 2008 to December 2016. The diagnosis of UC was established based on clinical, endoscopic, radiological, and histopathological findings in accordance with Lennard-Jones criteria. The severity of UC was evaluated using the Truelove and Witts Activity Index (Supplementary Table 1), and the location was assessed using colonoscopy at the initial diagnosis [20]. Individuals with any other immune-related diseases, including SLE, RA, MS, and autoimmune hepatitis were excluded. The demographic data of patients with UC and healthy controls are presented in Table 1. Besides, 34 patients with UC (18 females and 16 males, 40.17 ± 14.21 years) were selected and 36 patients with benign sigmoid polyps (20 females and 16 males, 41.02 ± 13.85 years) were simultaneously recruited as normal controls for comparing the colonic expression of *FOXP3*. Specimens of inflamed mucosa in the sigmoid were uniformly collected from 34 patients with UC during the colonoscopy examination. Similarly, specimens of normal sigmoid mucosa were obtained from each of the patients with benign sigmoid polyps (normal controls). All study subjects were recruited from The Second Affiliated Hospitals of Wenzhou Medical University in Zhejiang Province of southeast China.

### 2.2. Genomic DNA Extraction and Genotype Analysis

Approximately 1 mL of peripheral venous blood from each study individual was collected into ethylenediaminetetraacetic acid (EDTA) anticoagulated tubes. Genomic DNA was extracted from peripheral blood leukocytes using the DNeasy Blood and Tissue Kit (Qiagen GmbH, Hilden, Germany) and then stored at –20°C for further analysis.

Genotypes of *FOXP*3 (rs3761548, rs2232365, rs2294021, and rs3761547) were detected using the SNaPshot assays by Applied Biosystems (CA, USA) as described previously [21]. Briefly, the polymerase chain reaction (PCR) protocol was carried out in 10 *μ*L as follows: 1 *μ*L genomic DNA (10 ng), 1 *μ*L 10× PCR buffer with MgCl_2_ (Roche, Basel, Switzerland), 1 *μ*L dNTPs (Promega, WI, USA), 0.5 U of FastStart Taq DNA polymerase (Roche, Basel, Switzerland), and a defined concentration (0.1 *μ*mol/L) of each amplification primer. Primer sequences are provided in Supplementary Table 2. The amplification was performed in an ABI 9700 thermal cycler using an initial denaturation step at 95°C for 5 min, followed by 35 cycles of 95°C for 30 s, 65°C for 30 s, 72°C for 1 min, and finally 72°C for 10 min. The purification was conducted on 7.4 mL volume including 2 mL PCR product, 2 U of Exonuclease I (TaKaRa, Dalian, China), and 1.5 U of SAP (New England Biolabs, MA, USA), which were incubated for 80 min at 37°C and enzyme-inactivated at 85°C for 15 min. The SNaPshot multiplex sequencing reaction was performed in a final volume of 7 *μ*L containing 2 *μ*L PCR purification product, 1 *μ*L SNaPshot Multiplex Mix, 1 *μ*L 5× seq buffer, and 0.2 *μ*M of specific primer. The reaction was performed as follows: 96°C for 1 min, followed by 28 cycles of 96°C for 10 s, 52°C for 5 s, and 60°C for 30 s. Purification was carried out to degrade ddNTPs by adding 1 U of SAP, followed by an incubation at 37°C for 60 min and a step at 75°C for 15 min. The SNaPshot extension products were separated using capillary electrophoresis (3730xl Genetic Analyzer, Applied Biosystems) and analyzed using GeneMapper 4.0 (Applied Biosystems).

### 2.3. Real-Time Quantitative PCR (qPCR) Analysis for mRNA Expression of *FOXP3*

Total RNA was isolated from intestinal biopsies using the TRIzol™ Reagent (Thermo Fisher Scientific, MA, USA). The cDNA was prepared using the cDNA reverse transcription kit (Thermo Fisher Scientific, MA, USA). The primers were synthesized by the Sangon Biotech (Shanghai, China): 5′ AAGAGCTACGAGCTGCCTGAC 3′ (forward), 5′ GTAGTTTCGTGGATGCCACAG 3′ (reverse) for *β*-actin and 5′ GAAACAGCACATTCCAGAGTTC 3′ (forward) 5′ ATGGCCCAGCGGATGAG 3′ (reverse) for FOXP3. qPCR was performed in triplicate, using a Power SYBR Green PCR Master Mix (Applied Biosystems, CA, USA). PCR conditions for gene amplification began with 95°C for 10 min, followed by 40 cycles of 95°C for 15 s and 60°C for 1 min. Amplification was performed in a total volume of 25 *μ*L. The expression of *FOXP3* mRNA was normalized to the expression of *β*-actin. Relative gene expression was calculated using the ΔCt method.

### 2.4. Immunohistochemical Analysis for the Expression of FOXP3

The expression of FOXP3 in colonic tissues was measured using Immunohistochemical analysis as described previously [22]. Biopsy specimens were fixed in formalin and embedded in paraffin. These embedded specimens were cut into 2 mm sections and boiled in Tris-EDTA buffer (pH 9.0) in a cooker-cooler for 25 min. After blocking in endogenous peroxidase with 3% hydrogen peroxide solution at room temperature for 25 min, the slides were washed in phosphate-buffered saline (pH 7.4) three times for 5 min. The sections were incubated with 3% bovine serum albumin (Solarbio, Beijing, China) for 30 min to block unspecific antibody binding. Then, the sections were incubated with mouse anti-human FOXP3 antibody (Clone 236A/E, Abcam, Cambridge, UK; dilution 1 : 100) for 24 h at 4°C and with a secondary antibody (Invitrogen, CA, USA) for 30 min in a 37°C water bath. Detection was performed following treatment with immunoperoxidase using the EnVision system (Dako, Glostrup, Denmark). The expression of *FOXP3* was quantitatively evaluated using the Image-Pro Plus 6.0 analysis system (Media Cybernetics, MD, USA) by calculating the mean density, which was the integrated optical density (IOD) divided by the area of interest. Three fields of each slide at 200x magnification were randomly selected, and the mean density was obtained for further statistical analysis.

### 2.5. Statistical Analysis

All data were dealt with using SPSS 17.0 software for Windows (IL, USA). Patients with UC and healthy controls were divided into female and male groups separately for analyzing allelic and genotypic frequencies, as males are hemizygous for the four SNPs of *FOXP*3. An unconditional regression analysis was employed to address the association of *FOXP*3 polymorphisms with UC susceptibility and their influence on the clinical features of patients with UC after adjusting for age, gender, and smoking. Odds radios (ORs) and 95% confidence intervals (CIs) were applied for logistic regression analysis. Analyses for linkage disequilibrium (LD) and haplotype were conducted using the Haploview 4.2 software (MA, USA). The comparison of the expression level of FOXP3 in the colonic tissue was performed using the Student *t* test. The association between the colonic expression of *FOXP3* and the clinicopathological features of patients with UC, including gender, age, and disease severity, together with the four SNPs of *FOXP*3, was assessed using the linear regression analysis (stepwise). A two-tailed *P* value less than 0.05 was considered significant.

### 2.6. Ethical Considerations

This study protocol was in line with the Declaration of Helsinki and was approved by the ethics committee of The Second Affiliated Hospitals of Wenzhou Medical University. The informed consents were obtained from all study subjects.

## 3. Results

### 3.1. Comparison of *FOXP3* Polymorphisms between Patients with UC and Healthy Controls

As shown in Table 2, frequencies of variant alleles and genotypes of *FOXP3* (rs3761548, rs2232365, and rs2294021) were obviously higher in female patients with UC than in female healthy controls (all *P* < 0.05). When male patients with UC were compared with male healthy controls, the same results were obtained for variant alleles of the three SNPs (all *P* < 0.05) (Table 2). Patients with UC were divided into two subgroups based on the disease severity. The variant alleles and genotypes of *FOXP3* (rs3761548, rs2232365, and rs2294021) were more prevalent in female patients with severe UC than in female patients with mild and moderate UC (all *P* < 0.05) (Table 3). The allele differences in the three SNPs were also detected between the two subgroups in males (all *P* < 0.05) (Table 4).

**Figure 1:**
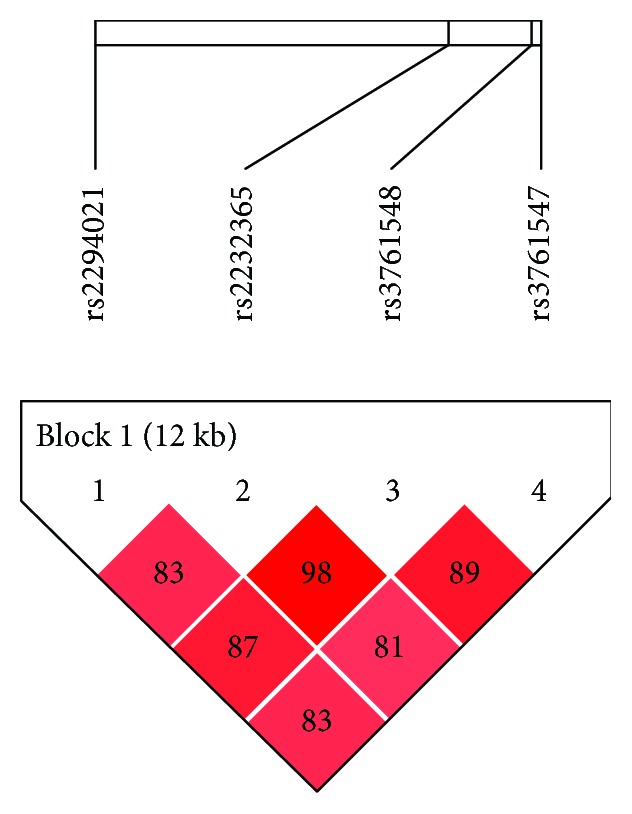
Linkage disequilibrium (LD) plot (obtained using Haploview 4.2 software) between *FOXP3* (rs2232365, rs2294021, rs3761548, and rs3761547) in female patients with ulcerative colitis (UC) and female controls. Each square plots a *D*′ value between a pair of polymorphic loci.

Since *FOXP3* is located on the X chromosome and males have only one copy of the X chromosome, their LD degree was assigned with 100% probability in this study. Thus, the haplotypes in males were sole and could be directly observed. For females, LD and haplotype were evaluated using the Haploview 4.2 software. As illustrated in Figure 1, the four polymorphic sites of rs3761548, rs2232365, rs2294021, and rs3761547 were also in a strong LD with each other in females. Unfortunately, no significant association of each haplotype with UC was observed in either females or males (all *P* > 0.05) (data not shown).

### 3.2. Comparison of the Expression of *FOXP3* between Inflamed Mucosa of Patients with UC and Normal Mucosa of Patients with Benign Sigmoid Polyps (Normal Controls)

**Figure 2:**
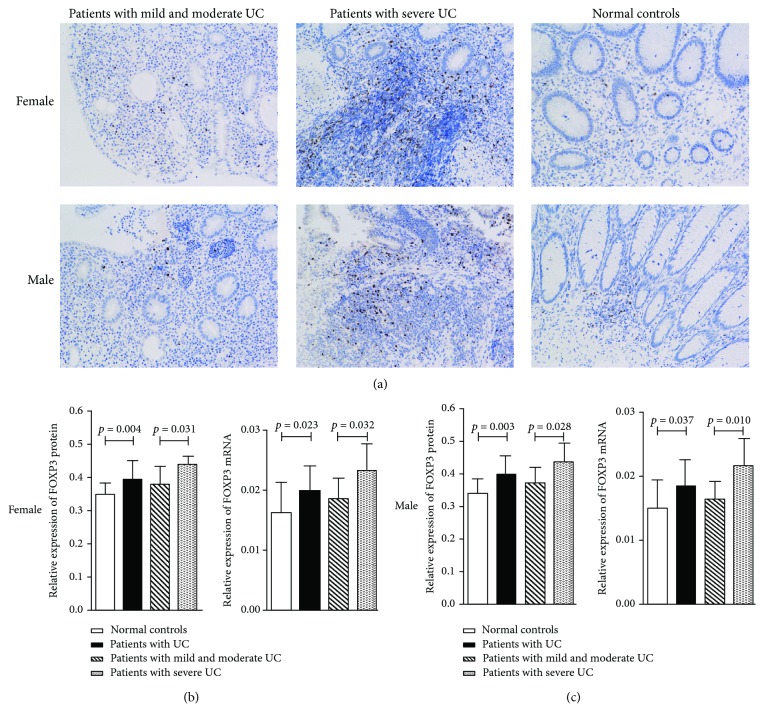
Expression of *FOXP3* in colonic tissues from patients with ulcerative colitis (UC) and normal controls. The protein and mRNA expression of *FOXP3* was evaluated quantitatively using the Image-Pro Plus 6.0 analysis system and the ΔCt method, respectively. Data are mean ± SD. (a) Immunohistological images of the expression of *FOXP3*. FOXP3 was detected in mucosa lamina propria with nuclear staining. Sections are shown at magnifications of ×200. (b) The expression of *FOXP3* in female subjects. (c) The expression of *FOXP3* in male subjects.

As depicted in Figure 2, the average expression of *FOXP3* mRNA and protein was upregulated in patients with UC compared with that in normal controls (in females or males) (females: mRNA: 0.020 ± 0.004*vs.*0.016 ± 0.005, *P* = 0.023; protein: 0.395 ± 0.055*vs.*0.349 ± 0.035, *P* = 0.004; males: mRNA: 0.018 ± 0.004*vs.*0.015 ± 0.004, *P* = 0.037; protein: 0.394 ± 0.059*vs.*0.336 ± 0.045, *P* = 0.003). Likewise, the average expression of *FOXP3* mRNA and protein was higher in the corresponding patients with severe UC compared with that in female and male patients with mild and moderate UC (females: mRNA: 0.023 ± 0.004*vs.*0.019 ± 0.003, *P* = 0.032; protein: 0.439 ± 0.026*vs.*0.378 ± 0.054, *P* = 0.031; males: mRNA: 0.022 ± 0.004*vs.*0.016 ± 0.003, *P* = 0.010; protein: 0.435 ± 0.057*vs.*0.370 ± 0.047, *P* = 0.028). However, there was no significant difference in colonic expression of *FOXP3* mRNA and protein between normal controls and patients with mild and moderate UC (in females or males) (all *P* > 0.05).

### 3.3. Effect of *FOXP3* Polymorphisms on the Colonic Expression of *FOXP3* in Patients with UC

**Figure 3:**
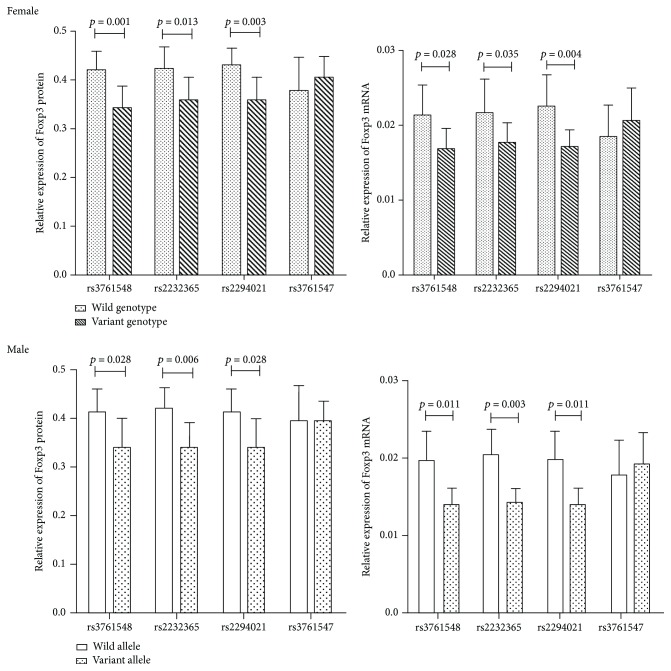
Expression of *FOXP3* protein and mRNA in patients with ulcerative colitis (UC) harboring the different alleles or genotypes of *FOXP3* (rs2232365, rs2294021, rs3761548, and rs3761547). The protein and mRNA expression of *FOXP3* was quantitatively evaluated using the Image-Pro Plus 6.0 analysis system and the ΔCt method, respectively. Data are mean ± SD. Variant genotypes: heterozygote and variant homozygote.

The average expression of *FOXP3* mRNA and protein in female patients carrying variant genotypes (CA + AA), (AG + GG), and (CT + TT) was downregulated compared with that in female patients with UC having wild homozygote (CC) of rs3761548, (AA) of rs2232365, and (CC) of rs2294021, respectively (mRNA: *P* = 0.028, 0.035, 0.004; protein: *P* = 0.001, 0.013, and 0.003, respectively). Similar conclusions were drawn for male patients with variant allele (A) of rs3761548, (G) of rs2232365, and (T) of rs2294021 compared with those with wild allele (C), (A), and (C), respectively (mRNA: *P* = 0.011, 0.003, and 0.011; protein: *P* = 0.028, 0.006, 0.028, respectively) (Figure 3).

Finally, a linear regression analysis was applied to address the association of the expression of *FOXP3* with the clinicopathological features of patients with UC. The included parameters were as follows: gender, age, severity and location of UC, and the four SNPs of *FOXP*3. Consequently, male gender (*β* = −0.341, *P* = 0.013), rs2294021 variation (*β* = −0.503, *P* = 0.001), and severe UC (*β* = 0.361, *P* = 0.013) were shown to be independently associated with the colonic mRNA expression of *FOXP3* in patients with UC (Table 5).

## 4. Discussion

The present study provided preliminary evidence that rs3761548, rs2232365, and rs2294021, rather than rs3761547, were likely to be risk loci affecting the predisposition of UC in the Chinese population. Theoretically speaking, rs3761548 and rs2232365 are located in the supposed DNA-binding sites of the promoter region of *FOXP3* and are therefore speculated to participate in the modulation of the expression of *FOXP3* [23]. More specifically, rs3761548 is located on *FOXP3* at position -3279, while rs2232365 is positioned at -924. Currently, both of them have been demonstrated to interfere with the interaction of some transcription factors, such as specificity protein 1 (Sp1), with the binding region within the *FOXP3* promoter, thereby having an impact on the transcriptional activity of *FOXP3* [24–26]. As for rs2294021, conclusive evidence exists that this SNP may indirectly influence the transcription of *FOXP3* mRNA by forming a LD with other functional loci of *FOXP3*, such as rs3761548 and rs5902434 [27]. Saxena et al. observed that *FOXP3* (rs3761548, rs2232365, rs2294021, and rs5902434) variations, together with the haplotype (A-G-C-ATT), could increase individual susceptibility to idiopathic recurrent miscarriages in an Indian population [27]. Another study in Poland Caucasians described that individuals harboring variant genotype and allele of rs3761548 or rs2232365, as well as haplotype AG constructed by the two SNPs, were more prone to RA [28]. Nevertheless, Gao et al. reported that genetic variation of rs3761548, but not of rs2232365, engendered a higher risk of psoriasis in the Chinese population [15]. The differences in the results of various studies implied the divergent impact of *FOXP3* polymorphisms on the immune-related diseases, especially in those with a different genetic background. Two likely explanations for this phenomenon were that (1) *FOXP3* polymorphisms may interact differentially with other genetic and environmental factors that change the biological context of *FOXP3* in different individuals and (2) there may be some loci which predispose individuals to disease in general and other loci that determine which class or more specifically which disease an individual is more likely to get.

Furthermore, the colonic expression of *FOXP3* mRNA and protein was found to be evidently upregulated in patients with UC and positively related to the severity of UC. These findings were basically consistent with the results of two previous studies by Iboshi et al. and Velikova *et al.*, in which the higher colonic expression of *FOXP3* was more prevalent in patients with UC compared with controls and was linked to a higher activity of the disease [29, 30]. Studies have demonstrated that FOXP3, mainly expressed in Tregs, is responsible for the differentiation, function, and phenotypic commitment of Tregs. Although FOXP3^+^ Treg accumulation in the inflamed mucosa of IBD has been observed in some studies, such an increase was not as apparent as in other intestinal inflammatory diseases, such as diverticulitis [5]. Concomitant to the evaluated frequency of colonic FOXP3^+^ Tregs, however, the percentage of circulating FOXP3^+^ Tregs was found to be obviously diminished. Therefore, this differential distribution of FOXP3^+^ Tregs between colonic mucosa and circulation could be partially attributed to an active recruitment and expansion of FOXP3^+^ Tregs in inflamed areas in an attempt to inhibit the intestinal inflammation. Unfortunately, the excessive intestinal inflammation seemed to be far beyond the compensation of FOXP3^+^ Tregs in patients with UC.

On the basis of the aforementioned findings, the present study further analyzed the influence of *FOXP3* polymorphisms on the colonic expression of *FOXP3* in patients with UC. As a consequence, the variations of rs3761548, rs2232365, and rs2294021, rather than rs3761547, were found to be related to the lower expressions of *FOXP3* in colonic tissues. Subsequently, the multivariate analysis suggested that the rs2294021 variation still had a negative impact on the colonic expressions of *FOXP3*, even after adjusting for the clinicopathological features of patients with UC. As mentioned earlier, rs3761548 and rs2232365 exist in the promoter region of *FOXP3*, and a strong LD exists between rs3761548, rs2232365, and rs2294021. To be more specific, rs3761548 is located in the core “GGGCGG” sequence of the putative binding site of some transcription factors within *FOXP3*, such as Sp1 [26]. Rs2232365 is mapped on *FOXP3* within the putative DNA-binding site of the transcription factor GATA-3 [31]. Since Sp1 and GATA-3 are essential for the modulation of the expression of *FOXP3* and Tregs function via interplay of the regulatory regions in *FOXP3*, it is believed that such two SNPs can affect the transcriptional activity of *FOXP3* and its protein expression [32, 33]. As for rs2294021, it has been suggested that this SNP may indirectly influence the transcription of *FOXP3* mRNA by forming a LD with other functional loci of *FOXP3*, such as rs3761548 [27]. Indeed, Zheng et al. reported that the rs3761548 mutation not only resulted in a higher risk of GD but also contributed to the reduced relative luciferase activity of the *FOXP3* promoter as well as the decreased mRNA expression of *FOXP3* in patients with GD [14].

It was worth mentioning that gender was an independent factor affecting the colonic expression of *FOXP3* in the present study. A similar result was also obtained from a study on rat kidney, where females had greater numbers of FOXP3^+^ Tregs than males [34]. Wildin and Freitas found that genes on the alternative X chromosome could interact with the *FOXP3* locus through its influence on X inactivation, thereby modifying the expression of *FOXP3* [35]. Moreover, the effects of gender-specific hormones (e.g., estrogen) or imprinting, an epigenetic phenomenon where allelic expression relies on the gender of the parent from whom this special allele is inherited, cannot be ignored [33, 35]. Interestingly, it has recently been shown that Y-chromosome-linked polymorphisms may differentially regulate the expression of X-linked genes [36]. Thus, hypothetically, *FOXP3* gene expression may be differentially modulated in females and males through some suppressive or activation mechanisms mediated by the Y chromosome.

In conclusion, the present study suggested that *FOXP3* (rs3761548, rs2232365, and rs2294021) variations increased the risk of UC and were related with the lower colonic expression of *FOXP3* in UC patients. Moreover, male gender, rs2294021 variation, and severe UC were independently related to the colonic expression of *FOXP3* in this cohort of Chinese patients with UC. Nevertheless, the cellular and molecular mechanisms by which *FOXP3* polymorphisms affect the colonic expression of FOXP3 are still not entirely clear. In addition, other types of colitis, such as infectious colitis and ischemic colitis, were not included in this study to exclude the impact of other inflammation on the colonic expression of FOXP3. Finally, the colonic expression of FOXP3 was not measured in all patients, but only in subgroups. It was impossible to confirm whether the subgroups used for examining the expression of *FOXP3* were representative of the full cohort of study subjects in terms of the influences of gender, *FOXP3* polymorphisms, or severity of UC in patients. Therefore, large-sample studies and subsequent *in vivo* and *in vitro* researches on the function of *FOXP3* polymorphisms are imperative.

## Figures and Tables

**Table 1:** Demographic characteristics of patients with ulcerative colitis (UC) and healthy controls (HC).

Characteristics	UC patients (*n* = 472)	HC (*n* = 525)	*P*
Gender (female/male)	232/240	270/255	0.473
Age (years) (mean ± SD)	41.22 ± 14.82	40.52 ± 15.33	0.768
Smoking			
Current or ex-smoker [frequency (%)]	189 (40.0)	201 (38.3)	0.570
Never smoked [frequency (%)]	283 (60.0)	324 (61.7)	
Extent of UC^∗^ [frequency (%)]			
Proctitis	106 (22.5)		
Left-sided	202 (42.8)		
Extensive	164 (34.7)		
Severity of UC [frequency (%)]			
Mild	166 (35.2)		
Moderate	151 (32.0)		
Severe	155 (32.8)		
Treatment [frequency (%)]			
Sulfasalazine/5-aminosalicylic acid	379 (80.3)		
Prednisone	183 (38.8)		
Antibiotics	136 (28.8)		
Immunosuppressive	12 (2.5)		
Colectomy	10 (2.1)		

^∗^Distal colitis was defined as disease lesion distal to spleen flexure, and extensive colitis was defined as disease lesion location beyond spleen flexure.

**Table 2:** Allelic and genotypic distribution of *FOXP*3 polymorphisms in patients with ulcerative colitis (UC) and healthy controls (HC) [frequency (%)].

*FOXP3*	Female HC (*n* = 270)	Female UC (*n* = 232)	Male HC (*n* = 255)	Male UC (*n* = 240)
rs3761548				
CC	172 (63.7)	126 (54.3)		
CA	77 (28.5)	73 (31.5)		
AA	21 (7.8)	33 (14.2)		
CA + AA	98 (36.3)	106 (45.7)^a^		
C	421 (78.0)	325 (70.0)	204 (80.0)	172 (71.7)
A	119 (22.0)	139 (30.0)^b^	51 (20.0)	68 (28.3)^g^
rs2232365				
AA	111 (41.1)	72 (31.0)		
AG	110 (40.7)	105 (45.3)		
GG	49 (18.2)	55 (23.7)		
AG + GG	159 (58.9)	160 (69.0)^c^		
A	332 (61.5)	249 (53.7)	174 (68.2)	138 (57.5)
G	208 (38.5)	215 (46.3)^d^	81 (31.8)	102 (42.5)^h^
rs2294021				
CC	80 (29.6)	50 (21.5)		
CT	108 (40.0)	93 (40.1)		
TT	82 (30.4)	89 (38.4)		
CT + TT	190 (70.4)	182 (78.5) ^e^		
C	268 (49.63)	193 (41.6)	113 (44.3)	83 (34.6)
T	272 (50.37)	271 (58.4)^f^	142 (55.7)	157 (65.4)^i^
rs3761547				
CC	27 (10.00)	16 (6.9)		
CA	63 (23.33)	58 (25.0)		
AA	180 (66.67)	158 (68.1)		
CA + AA	243 (90.00)	216 (93.1)		
C	117 (21.67)	90 (19.4)	52 (20.4)	50 (20.8)
A	423 (78.33)	374 (80.6)	203 (79.6)	190 (79.2)

^∗^All listed *P* and OR values were calculated for each SNP analyzed using logistic regression after adjusted for age, gender, and smoking. ^a^ *P* = 0.033,OR = 1.477,95% CI 1.032-2.112. ^b^ *P* = 0.004,OR = 1.513,95% CI 1.139-2.011. ^c^ *P* = 0.020,OR = 1.551,95% CI 1.073-2.244. ^d^ *P* = 0.012,OR = 1.378,95% CI 1.072-1.773. ^e^ *P* = 0.040,OR = 1.533,95% CI 1.020-2.304. ^f^ *P* = 0.011,OR = 1.383,95% CI 1.077-1.777. ^g^ *P* = 0.031,OR = 1.581,95% CI 1.043-2.397. ^h^ *P* = 0.014,OR = 1.588,95% CI 1.100-2.293. ^i^ *P* = 0.027,OR = 1.505,95% CI 1.047-2.164.

**Table 3:** Association of *FOXP*3 polymorphisms with the clinical features in female patients with ulcerative colitis (UC) [frequency (%)].

*FOXP*3	Severity of UC		Extent of UC	
	Mild and moderate (*n* = 155)	Severe (*n* = 77)	Distal colitis (*n* = 165)	Extensive colitis (*n* = 68)
rs3761548				
CC	96 (62.0)	30 (39.0)	86 (52.4)	40 (58.8)
CA + AA	59 (38.0)	47 (61.0)^a^	78 (47.6)	28 (41.2)
C	230 (74.2)	95 (61.7)	231 (70.4)	94 (69.1)
A	80 (25.8)	59 (38.3)^b^	97 (29.6)	42 (30.9)
rs2232365				
AA	62 (40.0)	10 (13.0)	46 (28.1)	26 (38.2)
AG + GG	93 (60.0)	67 (87.0)^c^	118 (71.9)	42 (61.8)
A	178 (57.4)	71 (46.1)	174 (53.1)	75 (55.1)
G	132 (42.6)	83 (53.9)^d^	154 (46.9)	61 (44.9)
rs2294021				
CC	47 (30.3)	3 (3.9)	32 (19.5)	18 (26.5)
CT + TT	108 (69.7)	74 (96.1)^e^	132 (80.5)	50 (73.5)
C	145 (46.8)	48 (31.2)	136 (41.5)	57 (41.9)
T	165 (53.2)	106 (68.8)^f^	192 (58.5)	79 (58.1)
rs3761547				
CC	12 (7.7)	4 (5.2)	11 (6.7)	5 (7.3)
CA + AA	143 (92.3)	73 (94.8)	153 (93.3)	63 (92.7)
C	60 (19.4)	30 (19.5)	63 (19.2)	27 (19.9)
A	250 (80.6)	124 (80.5)	265 (80.8)	109 (80.1)

All listed ORs, 95% CIs, and *P* value were calculated for each SNP analyzed using logistic regression after adjusted for age, gender, and smoking. ^a^ *P* = 0.001,OR = 2.549,95% CI 1.454-4.468. ^b^ *P* = 0.006,OR = 1.786,95% CI 1.182-2.698. ^c^ *P* < 0.001,OR = 4.467,95% CI 2.135-9.344. ^d^ *P* = 0.022,OR = 1.576,95% CI 1.069-2.325. ^e^ *P* < 0.001,OR = 10.735,95% CI 3.220-35.784. ^f^ *P* = 0.001,OR = 1.941,95% CI 1.291-2.917.

**Table 4:** Association of *FOXP*3 polymorphisms with the clinical features in male patients with ulcerative colitis (UC) [frequency (%)].

*FOXP3*	Severity of UC		Extent of UC	
	Mild and moderate (*n* = 162)	Severe (*n* = 78)	Distal colitis (*n* = 144)	Extensive colitis (*n* = 96)
rs3761548				
C	129 (79.6)	43 (55.1)	100 (69.4)	72 (75.0)
A	33 (20.4)	35 (44.9)^a^	44 (30.6)	24 (25.0)
rs2232365				
A	103 (63.6)	35 (44.9)	85 (59.0)	53 (55.2)
G	59 (36.4)	43 (55.1)^b^	59 (41.0)	43 (44.8)
rs2294021				
C	63 (38.9)	20 (25.6)	50 (34.7)	33 (34.4)
T	99 (61.1)	58 (74.4)^c^	94 (65.3)	63 (65.6)
rs3761547				
C	35 (21.6)	15 (19.2)	32 (22.2)	18 (18.7)
A	127 (78.4)	63 (80.8)	112 (77.8)	78 (81.3)

All listed ORs, 95% CIs, and *P* value were calculated for each SNP analyzed using logistic regression after adjusted for age, gender, and smoking. ^a^ *P* < 0.001,OR = 3.182,95% CI 1.768-5.726. ^b^ *P* = 0.006,OR = 2.415,95% CI 1.239-3.714. ^c^ *P* = 0.045,OR = 1.845,95% CI 1.014-3.357.

**Table 5:** Linear regression analysis for the related factors influencing the mRNA expression of *Foxp3* in UC patients.

Covariants	Unstandardized coefficients		Standardized coefficients	*t*	*P*	95% *CI* for *B*
	*B*	S.E.	*β*			
Age^a^	0.000	0.001	-0.009	-0.061	0.952	-0.003-0.002
Gender^b^	-0.003	0.001	-0.341	-2.654	*0.013*	-0.005--0.001
rs3761548^c^	-0.001	0.002	-0.057	-0.282	0.780	-0.004-0.003
rs2232365^c^	-0.001	0.001	-0.172	-1.067	0.295	-0.004-0.001
rs2294021^c^	-0.004	0.001	-0.503	-3.581	*0.001*	-0.007--0.002
rs3761547^c^	0.002	0.001	0.191	1.551	0.132	-0.001-0.004
Severity of UC^d^	0.003	0.001	0.361	2.640	*0.013*	0.001-0.006
Location of UC^e^	0.000	0.001	0.029	0.218	0.829	-0.002-0.003

^a^With reference to age < 40 years older. ^b^With reference to female. ^c^With reference to patients without any variant allele of *FOXP*3 (rs3761548, rs2232365, rs2294021, and rs3761547), respectively. ^d^With reference to mild and moderate UC. ^e^With reference to distal colitis. CI: confidence interval.

## Data Availability

The data used to support the findings of this study are available from the corresponding author upon request.

## Supplementary Materials

Supplementary Materials Supplementary Table 1: this index is used to evaluate the severity of UC as described in study subjects, in Materials and Methods. And for Supplementary Table 2, this is the description of primers of the FOXP3 gene used for PCR amplification and extension.

## References

[1] Grant C. R., Liberal R., Mieli-Vergani G., Vergani D., Longhi M. S. (2015). Regulatory T-cells in autoimmune diseases: challenges, controversies and--yet--unanswered questions. *Autoimmunity Reviews*.

[2] van Herk E. H., te Velde A. A. (2016). Treg subsets in inflammatory bowel disease and colorectal carcinoma: characteristics, role, and therapeutic targets. *Journal of Gastroenterology and Hepatology*.

[3] Tao J. H., Cheng M., Tang J. P., Liu Q., Pan F., Li X. P. (2017). Foxp3, regulatory T cell, and autoimmune diseases. *Inflammation*.

[4] Pereira L. M. S., Gomes S. T. M., Ishak R., Vallinoto A. C. R. (2017). Regulatory T cell and forkhead box protein 3 as modulators of immune homeostasis. *Frontiers in Immunology*.

[5] Maul J., Loddenkemper C., Mundt P. (2005). Peripheral and intestinal regulatory CD4+ CD25(high) T cells in inflammatory bowel disease. *Gastroenterology*.

[6] Li Z., Vermeire S., Bullens D. (2015). Restoration of Foxp3+ regulatory T-cell subsets and Foxp3- type 1 regulatory-like T cells in inflammatory bowel diseases during anti-tumor necrosis factor therapy. *Inflammatory Bowel Diseases*.

[7] Li Z., Arijs I., De Hertogh G. (2010). Reciprocal changes of Foxp3 expression in blood and intestinal mucosa in IBD patients responding to infliximab. *Inflammatory Bowel Diseases*.

[8] van Nieuwenhuijze A., Liston A. (2015). The molecular control of regulatory T cell induction. *Progress in Molecular Biology and Translational Science*.

[9] Oda J. M. M., Hirata B. K. B., Guembarovski R. L., Watanabe M. A. E. (2013). Genetic polymorphism in FOXP3 gene: imbalance in regulatory T-cell role and development of human diseases. *Journal of Genetics*.

[10] Pesenacker A. M., Cook L., Levings M. K. (2016). The role of FOXP3 in autoimmunity. *Current Opinion in Immunology*.

[11] Hori S., Nomura T., Sakaguchi S. (2003). Control of regulatory T cell development by the transcription factor Foxp3. *Science*.

[12] Huehn J., Beyer M. (2015). Epigenetic and transcriptional control of Foxp3+ regulatory T cells. *Seminars in Immunology*.

[13] Misra M. K., Mishra A., Pandey S. K., Kapoor R., Sharma R. K., Agrawal S. (2016). Association of functional genetic variants of transcription factor forkhead box P3 and nuclear factor-*κ*B with end-stage renal disease and renal allograft outcome. *Gene*.

[14] Zheng L., Wang X., Xu L. (2015). Foxp3 gene polymorphisms and haplotypes associate with susceptibility of Graves’ disease in Chinese Han population. *International Immunopharmacology*.

[15] Gao L., Li K., Li F. (2010). Polymorphisms in the FOXP3 gene in Han Chinese psoriasis patients. *Journal of Dermatological Science*.

[16] Jahan P., Sreenivasagari R., Goudi D., Komaravalli P. L., Ishaq M. (2013). Role of Foxp3 gene in maternal susceptibility to pre-eclampsia - a study from South India. *Scandinavian Journal of Immunology*.

[17] Hosseini A., Shanehbandi D., Estiar M. A. (2015). A single nucleotide polymorphism in the FOXP3 gene associated with Behçet’s disease in an Iranian population. *Clinical Laboratory*.

[18] In J. W., Lee N., Roh E. Y., Shin S., Park K. U., Song E. Y. (2016). Association of aplastic anemia and FoxP3 gene polymorphisms in Koreans. *Hematology*.

[19] Inoue N., Watanabe M., Morita M. (2010). Association of functional polymorphisms related to the transcriptional level of FOXP3 with prognosis of autoimmune thyroid diseases. *Clinical and Experimental Immunology*.

[20] Xia S. L., Lin X. X., Guo M. D. (2016). Association of vitamin D receptor gene polymorphisms and serum 25-hydroxyvitamin D levels with Crohn’s disease in Chinese patients. *Journal of Gastroenterology and Hepatology*.

[21] Xia S. L., Yu L. Q., Chen H. (2015). Association of vitamin D receptor gene polymorphisms with the susceptibility to ulcerative colitis in patients from Southeast China. *Journal of Receptor and Signal Transduction Research*.

[22] Hu D. Y., Zhang D. G., Zheng S. Z., Guo M., Lin X., Jiang Y. (2016). Association of ulcerative colitis with FUT2 and FUT3 polymorphisms in patients from Southeast China. *PLoS One*.

[23] Safari M. R., Ghafouri-Fard S., Noroozi R. (2017). FOXP3 gene variations and susceptibility to autism: a case-control study. *Gene*.

[24] Wu Z., You Z., Zhang C. (2012). Association between functional polymorphisms of Foxp3 gene and the occurrence of unexplained recurrent spontaneous abortion in a Chinese Han population. *Clinical & Developmental Immunology*.

[25] Jahan P., Tippisetty S., Komaravalli P. L. (2015). FOXP3 is a promising and potential candidate gene in generalised vitiligo susceptibility. *Frontiers in Genetics*.

[26] Song P., Wang X. W., Li H. X. (2013). Association between FOXP3 polymorphisms and vitiligo in a Han Chinese population. *British Journal of Dermatology*.

[27] Saxena D., Misra M. K., Parveen F., Phadke S. R., Agrawal S. (2015). The transcription factor forkhead box P3 gene variants affect idiopathic recurrent pregnancy loss. *Placenta*.

[28] Paradowska-Gorycka A., Jurkowska M., Felis-Giemza A. (2015). Genetic polymorphisms of Foxp3 in patients with rheumatoid arthritis. *The Journal of Rheumatology*.

[29] Iboshi Y., Nakamura K., Fukaura K. (2017). Increased IL-17A/IL-17F expression ratio represents the key mucosal T helper/regulatory cell-related gene signature paralleling disease activity in ulcerative colitis. *Journal of Gastroenterology*.

[30] Velikova T., Kyurkchiev D., Spassova Z. (2017). Alterations in cytokine gene expression profile in colon mucosa of inflammatory bowel disease patients on different therapeutic regimens. *Cytokine*.

[31] Naderi-Mahabadi F., Zarei S., Fatemi R. (2015). Association study of forkhead box P3 gene polymorphisms with unexplained recurrent spontaneous abortion. *Journal of Reproductive Immunology*.

[32] Watkin R. D., Nawrot T., Potts R. J., Hart B. A. (2003). Mechanisms regulating the cadmium-mediated suppression of Sp1 transcription factor activity in alveolar epithelial cells. *Toxicology*.

[33] Nie J., Li Y. Y., Zheng S. G., Tsun A., Li B. (2015). FOXP3(+) Treg cells and gender bias in autoimmune diseases. *Frontiers in Immunology*.

[34] Tipton A. J., Baban B., Sullivan J. C. (2012). Female spontaneously hypertensive rats have greater renal anti-inflammatory T lymphocyte infiltration than males. *American Journal of Physiology-Regulatory, Integrative and Comparative Physiology*.

[35] Wildin R. S., Freitas A. (2005). IPEX and FOXP3: clinical and research perspectives. *Journal of Autoimmunity*.

[36] Lemos B., Araripe L. O., Hartl D. L. (2008). Polymorphic Y chromosomes harbor cryptic variation with manifold functional consequences. *Science*.

