# Supplementary material for: Association of Ulcerative Colitis with FOXP3 Gene Polymorphisms and Its Colonic Expression in Chinese Patients
Source: Gastroenterol Res Pract. 2019 Feb 24;2019:4052168. doi: 10.1155/2019/4052168 (PMC6409000; doi:10.1155/2019/4052168)
Supplement: Supplementary Materials — Supplementary Table 1: this index is used to evaluate the severity of UC as described in study subjects, in Materials and Methods. And for Supplementary Table 2, this is the description of primers of the FOXP3 gene used for PCR amplification and extension. [file 4052168.f1.doc]

**Supplementary Table 1. The Truelove and Witts Activity Index**

|  | **Mild** | **Moderate** | **Severe** |
| --- | --- | --- | --- |
| Bowel movements (no. per day) | Fewer than 4 | 4–6 | 6 or more plus at least one of the features of systemic upset (marked with ^*^ below) |
| Blood in stools | No more than small amounts of blood | Between mild and severe | Visible blood |
| Pyrexia (temperature ≥ 37.8°C) ^*^ | No | No | Yes |
| Pulse rate ≥ 90 bpm ^*^ | No | No | Yes |
| Anaemia ^*^ | No | No | Yes |
| ESR (mm/hour) ^*^ | 30 or below | 30 or below | Above 30 |

ESR: Erythrocyte sedimentation rate

**Supplemental Table 2**. Amplification and extension primers of the *FOXP*3 gene

| *FOXP*3 | Amplification primer (5'→3') | Size (bp) | Extension primer (5'→3') | Size (bp) |
| --- | --- | --- | --- | --- |
| rs3761548 | F:TCTCTGGTCTTCAATTTGCCCTTCTAC  R:CTCTCTTGCTCGCTCTTTGTGTGT | 202 | **35T**-TCTGGCTCTCTCCCCAACTG | 44 |
| rs2232365 | F:GAGGCGAGTCCAGGAGTGTGAT  R:AGAGGAGAAGGAGTGGGCATTTGA | 194 | **24T**-GTGACAGAGAGGAGGAGAGA | 47 |
| rs2294021 | F:GGTACACATGAGGACCCTCCACTG  R:CCCAGCCAGCCAATTAGCAGATG | 249 | **27T**-GATCTGGCAGACACCATGGC | 41 |
| rs3761547 | F:CCAACGTGTGAGAAGGCAGAAGG  R:GCAGCGGCAGAGTTGAAATCCA | 160 | **21T**-GTCCCCTGGATAGAGGGGCA | 55 |
